# Elucidating the Activation Mechanism of the Insulin-Family Proteins with Molecular Dynamics Simulations

**DOI:** 10.1371/journal.pone.0161459

**Published:** 2016-08-22

**Authors:** Anastasios Papaioannou, Serdar Kuyucak, Zdenka Kuncic

**Affiliations:** 1 Charles Perkins Centre, University of Sydney, Sydney, NSW, Australia; 2 School of Physics, University of Sydney, Sydney, NSW, Australia; University of Lincoln, UNITED KINGDOM

## Abstract

The insulin-family proteins bind to their own receptors, but insulin-like growth factor II (IGF-II) can also bind to the A isoform of the insulin receptor (IR-A), activating unique and alternative signaling pathways from those of insulin. Although extensive studies of insulin have revealed that its activation is associated with the opening of the B chain-C terminal (BC-CT), the activation mechanism of the insulin-like growth factors (IGFs) still remains unknown. Here, we present the first comprehensive study of the insulin-family proteins comparing their activation process and mechanism using molecular dynamics simulations to reveal new insights into their specificity to the insulin receptor. We have found that all the proteins appear to exhibit similar stochastic dynamics in their conformational change to an active state. For the IGFs, our simulations show that activation involves two opening locations: the opening of the BC-CT section away from the core, similar to insulin; and the additional opening of the BC-CT section away from the C domain. Furthermore, we have found that these two openings occur simultaneously in IGF-I, but not in IGF-II, where they can occur independently. This suggests that the BC-CT section and the C domain behave as a unified domain in IGF-I, but as two independent domains in IGF-II during the activation process, implying that the IGFs undergo different activation mechanisms for receptor binding. The probabilities of the active and inactive states of the proteins suggest that IGF-II is hyperactive compared to IGF-I. The hinge residue and the hydrophobic interactions in the core are found to play a critical role in the stability and activity of IGFs. Overall, our simulations have elucidated the crucial differences and similarities in the activation mechanisms of the insulin-family proteins, providing new insights into the molecular mechanisms responsible for the observed differences between IGF-I and IGF-II in receptor binding.

## Introduction

Insulin and insulin-like growth factors (IGFs) are proteins that share high sequence and structural homology, but have different cellular origins and different mechanisms of processing, secretion and circulation in the blood. In mammals, insulin is synthesized in the pancreas within the β-cells of the Islets of Langerhans, whereas IGFs are produced primarily by the liver, although other tissues are also involved [1]. While insulin regulates glucose levels in the blood, insulin-like growth factor I (IGF-I) helps to promote normal bone and tissue growth and development, and insulin-like growth factor II (IGF-II) plays a key role in proliferation and differentiation of fetal cells in many different tissues. Despite the fact that insulin is a two chain protein and IGFs are single chain proteins, there is remarkable overlap between the structures of the A and B chains of insulin and parts of the IGFs (hence the name insulin-like).

Insulin family proteins bind to their own receptors with high affinity, but IGF-II, in contrast to IGF-I, has also been reported to bind with high affinity to the A isoform of the insulin receptor (IR-A), which lacks the alternatively spliced exon 11 [2–5]. Cancer cell proliferation, survival and migration are promoted upon IGF-II binding to the IR-A, which activates unique signaling pathways different from those activated by insulin binding [6–13]. Previous studies have shown that several cancer cells, such as breast cancer, colorectal cancer and sporadic adrenocortical tumors, express both IGF-II and IR-A [7–9, 14–16]. This suggests that, while developmental growth is mediated through the insulin-like growth factor 1 receptor (IGF-1R), IGF-II/IR-A signaling can provide an additional or alternate pathway to stimulate cell growth, allowing cancer cells to become resistant to treatments targeting the IGF-1R [6,7,10]. Elucidating the way in which the IGFs interact with and bind to the IR-A is thus of significant interest in cancer biology for identifying a potential therapeutic target for pharmacological applications.

Previous experimental studies have revealed the critical role of the C domain and, to a lesser extent, the D domain of the IGFs for insulin receptor (IR) and IGF-1R binding specificity and activation [1,4,17–19]. Dentley et al have shown that IGF-I and IGF-II chimeras with swapped C and/or D domains perform with similar binding affinities to those of the donor molecules, and an IGF-I chimera comprising IGF-II C domain induced autophosphorylation of the IR-A and IR-B (B isoform of the insulin receptor) and activated signaling pathways in a similar way to IGF-II [3]. These results also highlight the opposing roles of the C and D domain sizes in regulating the binding specificity to the IR and IGF-1R, i.e. a smaller C domain leads to a higher binding affinity to IR, while a larger C domain leads to a higher binding affinity to IGF-1R [3]. Although the smaller size of the IGF-II C domain displays higher binding affinity to the IR-A, a larger C domain such as in IGF-I can also be accommodated in the IR-A binding pocket, as shown in a study where an IGF-I analogue comprising four insulin substitutions in the A and B chains exhibited higher affinity to the IR-A [20]. The important role of the C domain has been further supported in a study by Menting et al, who suggested that the difference in the binding affinities of IGFs for IR is at least in part due to the different sizes of the C domain, which in IGF-II can be accommodated relatively easily in the volume between the leucine-rich repeat domain (L1-β2) surface and the cysteine-rich (CR) domain of the IR [18]. Menting et al’s results indicate that IGFs bind to their receptor in a similar manner to insulin, with respect to binding site 1 [18]. Mutagenesis studies have revealed that when IGF-I binds to IGF-1R, it interacts with the L1 domain, the alpha-CT (αCT) peptide and the CR domain. In contrast, IGF-II interacts only with the L1 domain and the αCT peptide when it binds to IGF-1R, suggesting that IGF-I and IGF-II utilize different mechanisms to bind to the same receptor [21]. Earlier work of Bayne et al indicated that the purpose of the single chain structure of IGFs is to maintain selectivity rather than maximizing IGF receptor affinity [22]. Clearly, the C domain of IGFs plays a critical role in receptor specificity and binding affinity. To date however, the activation mechanisms of IGFs, and in particular of IGF-II, upon binding to the IR-A, still remain unknown.

The activation mechanism of insulin, in contrast, has been extensively studied [23–28]. In our recent study on insulin [23], we used molecular dynamics (MD) simulations to elucidate the conformational changes that occur upon insulin activation. In the present study, we performed MD simulations of wild-type (WT) IGF-I and IGF-II, to gain new insights into the critical differences and similarities between their structures that determine their activation mechanism and their specificity to the IR-A. These new results were compared to our previous results on insulin [23]. The activation dynamics and energetics of the IGFs were studied by analyzing data from long MD simulations. The stability and activity of the insulin-family proteins were examined and compared by investigating the hinge and the hydrophobic interactions in the core, as well as the probabilities of the different conformations of the activated states. Finally, water molecules in the immediate vicinity of the core and the network of the hydrogen bonds were studied to elucidate the mechanism that triggers the transition from the inactive to the active conformations.

## Methods

### Structure of the insulin-family proteins

**Fig 1:**
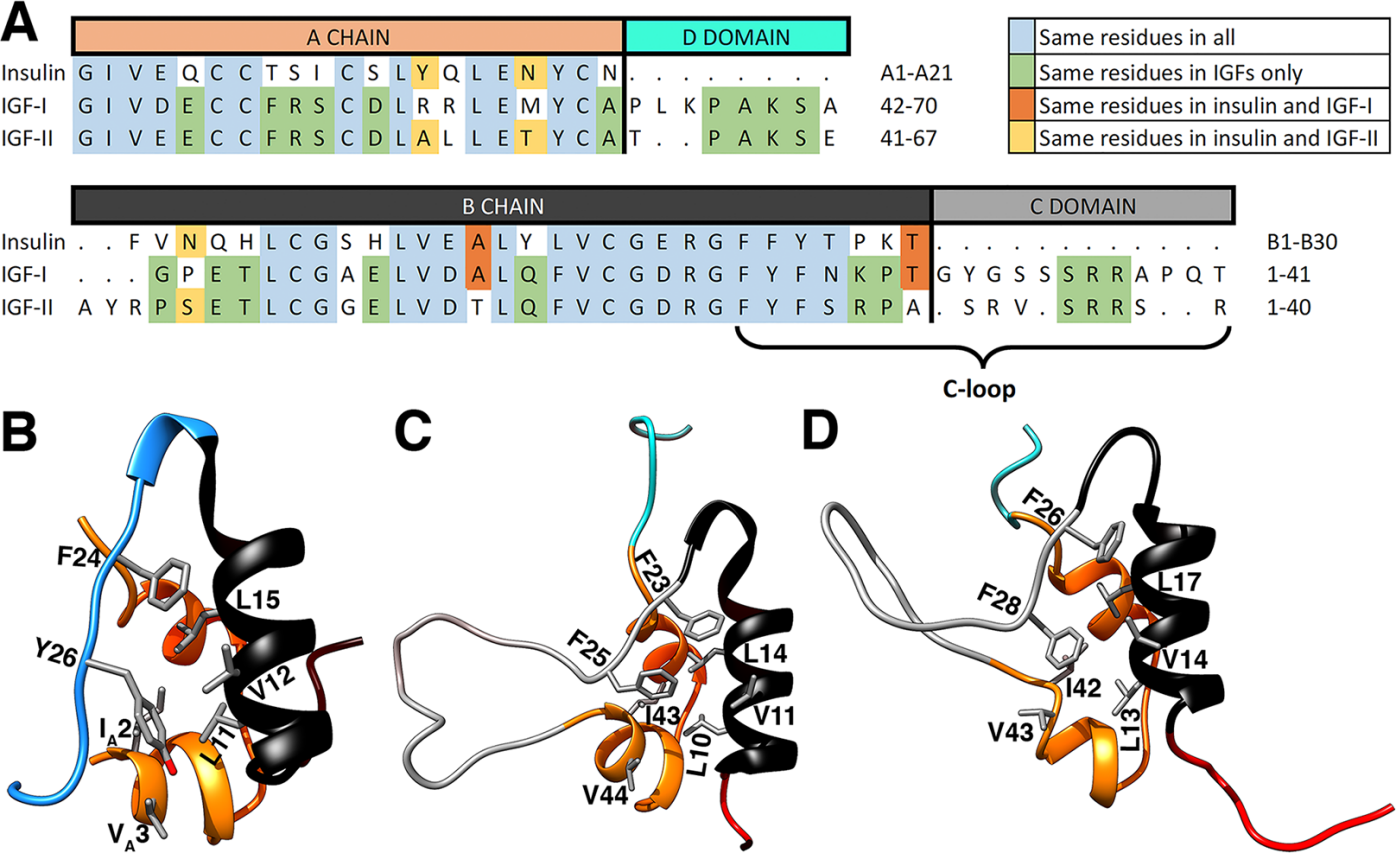
Sequence and structural comparison of insulin-family proteins, insulin, IGF-I and IGF-II. (A) Sequence comparison: Residual similarity between all three proteins is indicated with light blue, between IGFs in green, between insulin and IGF-I in orange, and between insulin and IGF-II in yellow. Amino-acids that belong to the same amino acid group were taken as similar. (B-D) Structures of (B) insulin, (C) IGF-I, and (D) IGF-II, comparing the residues involved in the hydrophobic core. In insulin, the B chain is shown in black, the BC-CT in light blue and the A chain in orange. In IGFs, the C loop (the C domain including the part of the B domain after the hinge residue) is shown in grey, the B domain N-terminal in red, rest of the B domain in black, the A domain in orange and the D domain in cyan. The spatial positions of the residues in the hydrophobic core are very similar in all three proteins.

Insulin consists of two chains, namely, a 21 amino acid chain A made of two α-helices, and a 30 amino acid chain B comprising a central α-helix (Fig 1). IGFs have substantial homology and structural similarity to insulin but are made of a single chain (S1 Table and Fig 1). To emphasize this homology, IGFs are represented in four domains as B, C, A and D (from N to C terminal), where the domains B and A correspond to the chains B and A in insulin (see the sequence alignment in Fig 1*A*). The C domain is a loop that interconnects the B and A domains, and the D domain is an extension of the A domain. The C domains exhibit the least homology between the IGFs, and these structural differences will be shown to have a major impact on their activation mechanisms. Because the partition of the IGFs into four domains is based on the insulin structure, it does not provide a very useful platform for discussing the activation mechanism in IGFs. To facilitate the description of activation, we define a C loop, which starts from the conserved hinge residue in the B domain (F23 in IGF-I and F26 in IGF-II) and continues until the end of the C domain (Fig 1).

Previous studies on insulin activation elucidated the critical behavior of the BC-CT section, which opens away from the B chain α-helix to expose its hydrophobic core during the activation process [23–25]. The hydrophobic core and its interactions thus play a critical role in the activation of insulin [23–25]. The hydrophobic core of insulin consists of the residues L11, V12, L15, F24 and Y26 from the B chain and I_A_2 and V_A_3 from the A chain. IGFs also comprise a hydrophobic core in their structures. The residues that are involved in the hydrophobic interactions of IGFs are located at the same spatial positions as in insulin, with the only difference being their sequence position as shown in Fig 1. In IGF-I, the hydrophobic core residues are L10, V11, L14, F23, F25, I43, and V44, while in IGF-II they are L13, V14, L17, F26, F28, I42, and V43. It is seen from Fig 1 that tyrosine in the Y26 residue of the hydrophobic core of insulin, which has been shown to play a critical role in the activation process and activity of insulin [23, 29–32], is replaced with phenylalanine in the F25 and F28 residues of IGF-I and IGF-II, respectively. The residue F24 in insulin has also been extensively studied and appears to play a hinge role in the activation of the BC-CT [23, 25, 33]. The corresponding residues in IGF-I and IGF-II (F23 and F26) remain the same.

### Modeling of IGFs

The MD simulations were performed using the crystal structure of the human IGF-I at 2 Å resolution (PDB ID: 1GZR [1]). No crystal structure is available for IGF-II. Therefore, its structure was derived from the NMR complex structure of IGF-II bound to the IGF 2 receptor (IGF2R) domain 11 (PDB ID: 2L29 [34]). There are 20 NMR conformations for the IGF-II/IGF2R complexes, and a representative structure was chosen for the simulations.

The VMD software [35] was used to build and prepare all the simulation systems. The following protocol was employed for the minimization and equilibration of IGF-I and IGF-II systems. IGF-I and IGF-II were solvated with approximately 5000 and 4200 water molecules, respectively, in a water box with periodic boundary conditions. The systems were neutralized and ionized to 0.1 M by randomly placing sodium and chloride ions in water. The (x, y, z) dimensions of the simulation box was (55, 58, 55) Å for IGF-I and (58, 62, 41) Å for IGF-II. The simulation systems were then energy minimized followed by equilibration.

The systems were equilibrated in two stages. During the first stage, the protein atoms were restrained and the system was equilibrated using 1 atm pressure coupling until the correct water densities were obtained. The second stage was slightly different for IGF-I and IGF-II, because the structure of IGF-II was derived from an NMR complex structure and the protein stability had to be maintained during the equilibration. In IGF-I, the restraints, applied on the side chain and backbone atoms, were relaxed by gradually reducing them from k = 30 to 0.1 kcal/mol/Å^2^. In IGF-II, a similar relaxation process was employed, but starting from k = 10 kcal/mol/Å^2^ and gradually reducing to k = 0.1 kcal/mol/Å^2^ using a smaller step-size and also examining the stability of the protein in each step of the process. In the last step of the equilibration, there were no restraints on the protein atoms, and the system was ready for production in MD simulations. The RMSD (Root Mean Square Deviation) was used to monitor the relaxation and equilibration of the protein.

### MD Simulations of IGFs

MD simulations were carried out with the NAMD package [36] using the CHARMM36 force field [37,38]. The NpT ensemble was employed with periodic boundary conditions in combination with the Particle Mesh Ewald (PME) method for calculating the long range electrostatic interactions in the periodic system. Langevin coupling with a damping coefficient of 5 ps^-1^ was employed to maintain a constant temperature at 300 K and pressure at 1 atm. The Lennard-Jones interactions were cut off at 12 Å and a switching function was used to smoothly diminish the interactions towards the cut-off distance. The cut-off distance of the non-bonded interactions, i.e. electrostatic and van der Waals interactions between atoms, was set at 13.5 Å using an update frequency of 1 ps. A time step of 2 fs used in all simulations and the trajectory data were recorded at every 15 ps.

The MD simulations were performed for almost 1μs in both IGF-I (870 ns) and IGF-II (900 ns). In both cases, the simulations were split into six shorter ones to improve sampling (6 short simulations of 145 and 150 ns in IGF-I and IGF-II, respectively) by using different relaxation times for the starting configuration in each case, i.e. 250, 300, …, 500 ps. The molecular graphics and analyses were performed using VMD [35] and UCSF Chimera packages [39]. The statistical analyses, discussed in the Results/Discussion section, are based on the long MD simulations of IGF-I, IGF-II and our previous study of WT insulin [23].

To check the results obtained from the MD simulations of IGF-II using the 2L29 structure, we additionally performed MD simulations of IGF-II using the solution structure of IGF-II (PDB ID: 1IGL [40]) and the crystal structure of IGF-II from the complex structure of human IGF2R domains 11–13 bound to IGF-II (we modelled the missing part of the C domain) (PDB ID: 2V5P [41]). The results from all three simulations of IGF-II were found to be consistent. Therefore, only the data obtained from the long MD simulation of IGF-II using the 2L29 structure are presented here.

### Potential of Mean Force (PMF) calculation

The PMF is used to examine the variation of the free energy of a system with respect to a specific reaction coordinate in MD simulations. The energetics of the activation process of IGFs were characterized by choosing the distance between the C_α_ atoms of the critical residue of the C loop from its closest partner in the B domain α-helix, namely F25(C_α_)-V11(C_α_) in IGF-I and F28(C_α_)-V14(C_α_) in IGF-II, and the distance between the C_α_ atoms of the next residue and its closest partner at the intersection of the A and C domains, namely N26(C_α_)-G42(C_α_) in IGF-I and S29(C_α_)-R40(C_α_) in IGF-II. These distances were chosen because they provide the best description for the opening process of the C loop. Because adequate sampling of IGFs was obtained from the MD simulations, the PMF was calculated directly from the probability density function (PDF) data employing the Boltzmann equation,

W=−kTln(ρρ0) (1)

where *T* is the system temperature (*T* = 300 K), *ρ* is the density obtained from the distance distributions, and *ρ*_*0*_ is a reference density, which we choose as the maximum density for convenience. In the PDF calculation, we set the number of bins to the square root of the number of data, i.e. 170 bins. Using the PMF information, we were able to estimate the change in the system’s free energy when the protein activates in all three insulin-family proteins.

**Fig 2:**
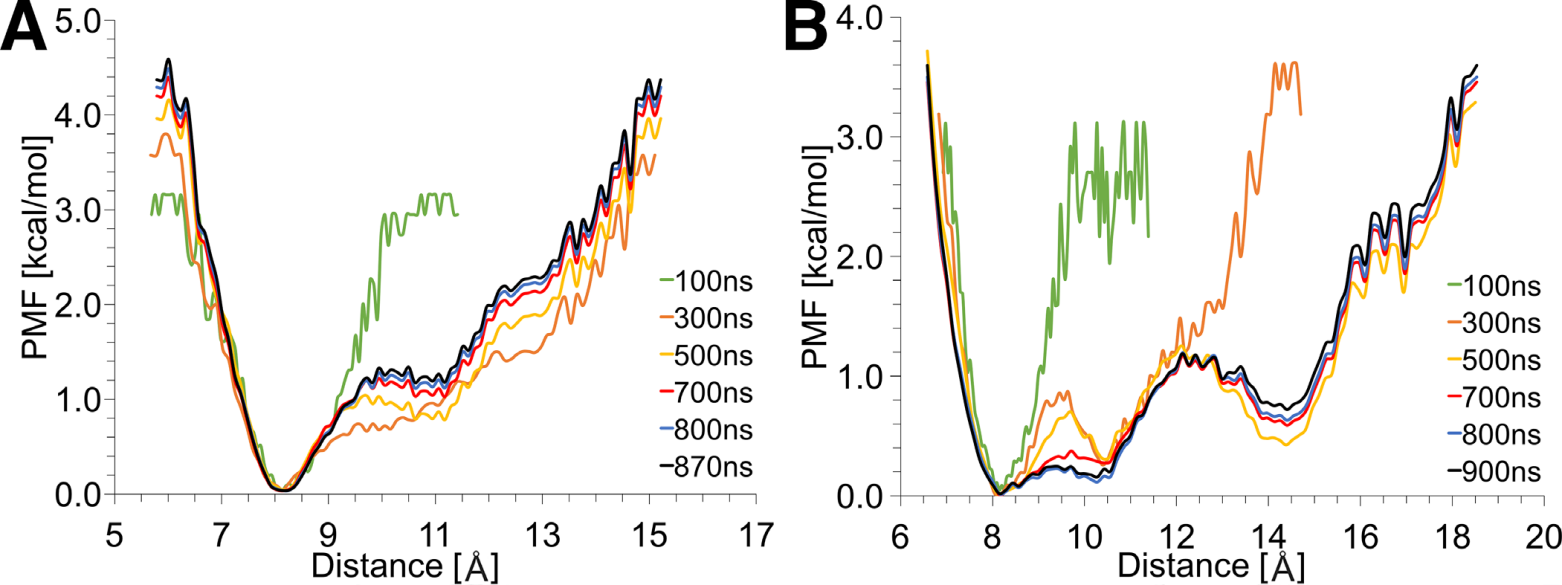
**Convergence analysis of the PMF calculation over the simulation time in (A) IGF-I and (B) IGF-II**.

We also performed a convergence analysis of the PMF calculations over the simulation time as shown in Fig 2. For both IGFs, the PMF curves begin to acquire their final form after ≈500 ns and converge after 700 ns of simulation time. This confirms that the total MD simulation times (870 ns and 900 ns for IGF-I and IGF-II, respectively) are sufficiently long for reliable PMF analysis as described above.

## Results and Discussion

### Comparison of the activation dynamics and energetics of the insulin-family proteins

**Fig 3:**
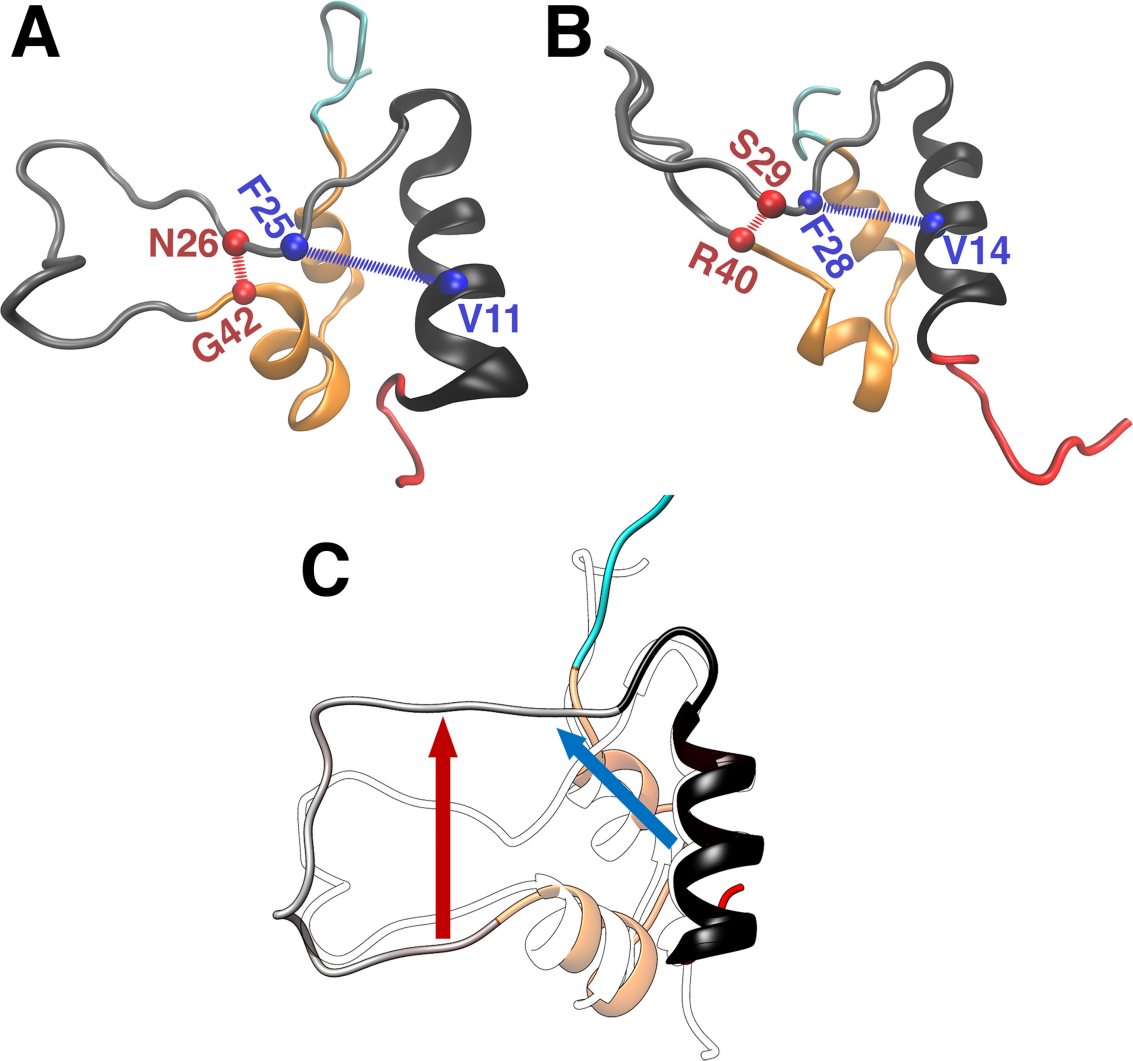
**Illustration of the distances of the C**_**α**_ **atoms used in describing the core opening (blue spheres and lines) and the C domain opening (red spheres and lines) in (A) IGF-I and (B) IGF-II and (C) the directions of the two openings of the C loop in IGF-I (a similar behavior occurs in IGF-II).** (A, B) The distance of the C_α_ atoms between the critical residue F25/F28 (where numbering refers to the IGF-I/IGF-II residue) and its closest partner V11/V14 in the B domain α-helix was chosen as the criterion for the core opening, whilst the distance of the C_α_ atoms between N26/S29 (the residue after the critical one) and its closest partner G42/R40 was chosen as the criterion for the C domain opening. (C) The closed state of IGF-I is shown in transparent outline while the open state is shown in colors as in (A).

Insulin activation is associated with the opening of the BC-CT away from its hydrophobic core [23–25]. In IGFs, this role is taken over by the C loop (Fig 1), whose opening triggers activation of IGFs. Inspection of the trajectories of the long MD simulations (cf. S1 and S2 Figs) revealed the existence of two distinct opening locations of the C loop in IGFs, as shown in Fig 3*C*. Interestingly, the first opening is very similar to insulin, i.e. the upper flank of the C loop (residues F23-F25 and F26-F28 in IGF-I and IGF-II, respectively), moves away from the B domain α-helix (hydrophobic core). In the second opening, the remaining part of the upper flank of the C loop (residues N26-G30 and S29-S33 in IGF-I and IGF-II, respectively) moves away from the lower flank of the C loop contiguously from the first C loop opening. Henceforth, we will refer to the first opening as the core opening, and the second one as the C domain opening.

To elucidate the behavior of the core and C domain openings in the IGFs, we calculated the distances between the C_α_ atoms of the critical residues (F25/F28 in IGF-I/IGF-II) and their closest partners in the B domain α-helix (V11/V14), and the distances between the next C_α_ atoms (N26/S29) and their closest partners at the intersection of the A and C domains (G42/R40) (see Fig 3*A* and 3*B*). The time series of the distances representing the core opening in insulin, IGF-I and IGF-II are shown in S1 Fig, and the distances representing the C domain opening in IGFs are shown in S2 Fig. Interestingly, all three proteins exhibit the same stochastic behavior during the MD simulations, with no pattern in the timing or frequency of occurrence of the core and C domain openings. This indicates that the C loop makes random excursions away from the hydrophobic core, similar to that found for the core opening in insulin [23].

**Fig 4:**
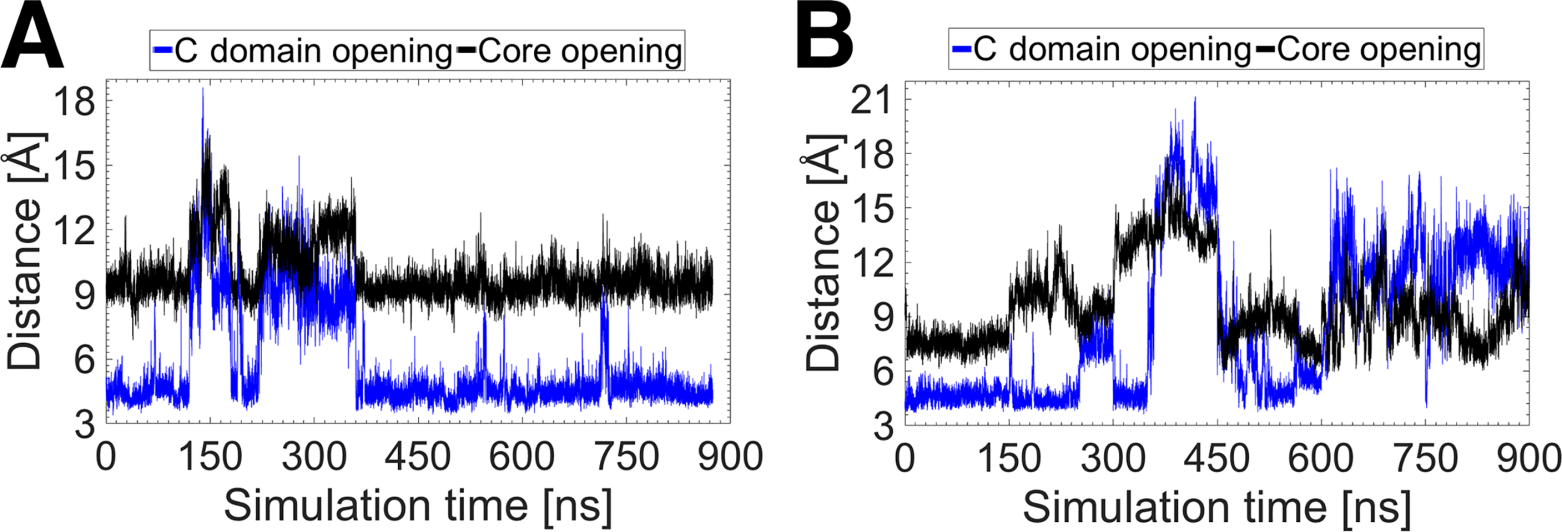
**Temporal correlation between the core and C domain openings in (A) IGF-I and (B) IGF-II.** The core and C domain openings are shown in black and blue, respectively.

The temporal profiles of the core and C domain openings in the IGFs are shown in Fig 4. In IGF-I, the two openings are highly correlated, occurring at the same time and with the same frequency. This coordinated, collective dynamics suggests that the C loop behaves as a single unified domain during the activation of IGF-I. This further suggests that the transition from the closed to the open state may be energetically difficult for IGF-I as the whole C loop has to open simultaneously during the activation process. In contrast, the core and C domain openings in IGF-II appear to be occurring independently of each other, as inferred from the uncorrelated times and frequencies. The independent movement of the aromatic triplet (F26-Y27-F28) from the rest of the C loop confers the C loop additional flexibility. As a result, the C loop can behave as a relatively hyperactive domain. The difference in the activation mechanisms of IGF-I and IGF-II is expected to affect their binding mechanisms, which is supported by experimental studies [21]. The different activation dynamics of the IGFs is also likely to contribute to their different specificity and sensitivity to IR-A. The independent occurrence of the two openings makes it energetically easier for IGF-II to expose first the hydrophobic core and the primary part of the C loop upper flank to the hydrophobic pocket of the L1 domain of the IR-A, and then to accommodate the remaining part of the C loop upper flank to the upper part of the L1 domain, avoiding any steric clashes with the αCT peptide of the IR-A, and *vice versa*. Overall this is expected to result in high binding affinity for IGF-II, but not for IGF-I.

**Fig 5:**
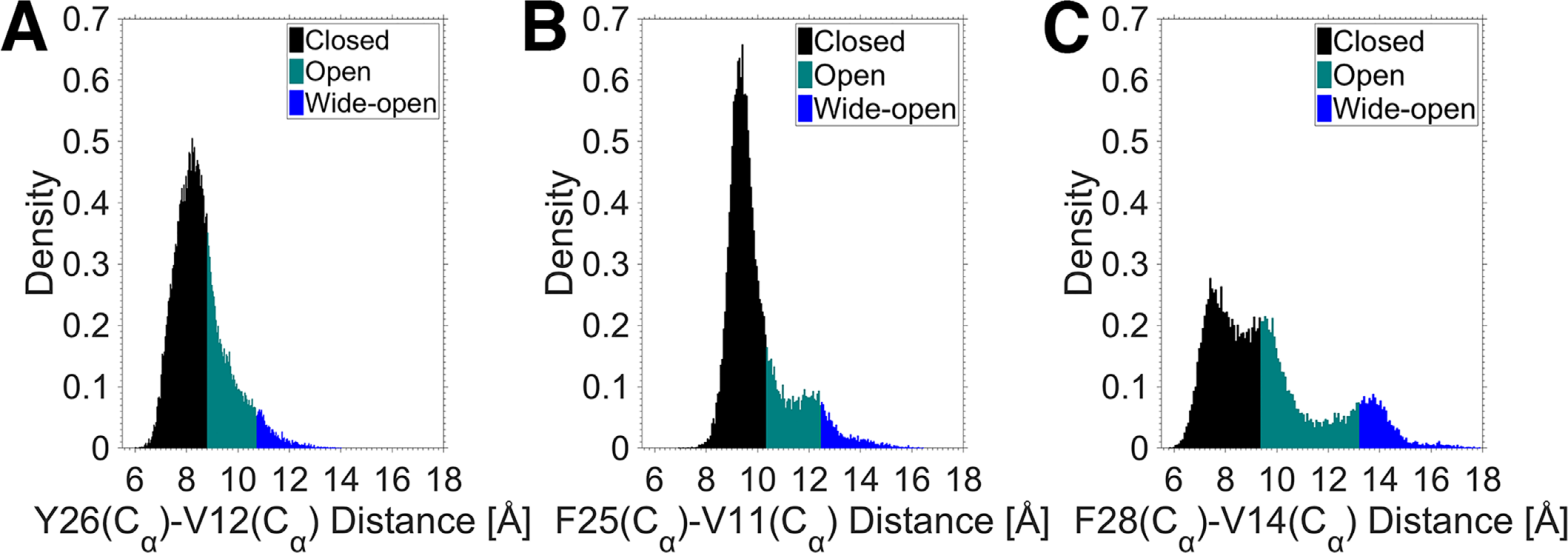
**Distribution of the distance representing the core opening in (A) insulin, (B) IGF-I and (C) IGF-II.** The differently colored histograms represent the three different conformations of the proteins, with black, cyan and blue denoting the closed, open and wide-open conformations, respectively.

**Fig 6:**
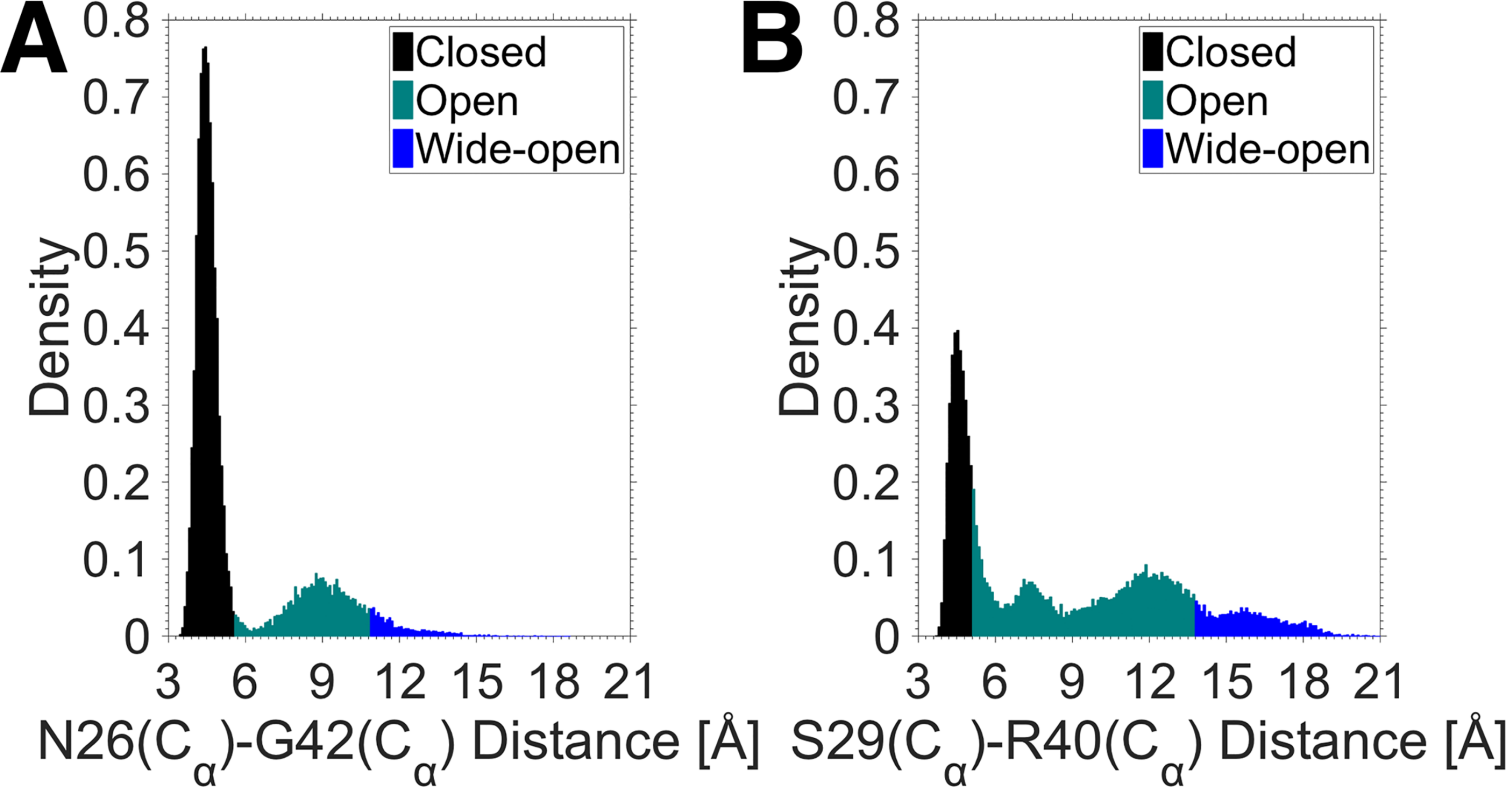
**Distribution of the distance representing the C domain opening in (A) IGF-I and (B) IGF-II.** Similar to Fig 4, the different histogram colors correspond to the different conformations of the IGFs.

To investigate the frequency of the core and C domain openings further, the distances, derived from the long MD simulations, were converted into probability densities. The probability density functions (PDFs) for the core and C domain openings are shown in Figs 5 and 6, respectively. The frequency histograms in Figs 5 and 6 represent the probability of a distance occurring over the course of the MD simulations. The different colors of the histograms define three distinct conformations observed during the MD simulations, similar to that found for insulin in our previous study [23]. The closed conformation corresponds to the inactive state of the proteins, the open conformation corresponds to the transition from the inactive to the active state when water molecules enter the hydrophobic core and break the interactions, and the wide-open conformation is defined as the open conformation that fits the receptor binding interface. The transition threshold from the closed to the open conformation in the core opening was determined by the number of water molecules in the hydrophobic core, i.e. none implies a closed conformation, and one or more water molecules, an open conformation. On this basis, a distance less than 8.7, 10.3 and 9.3 Å indicates a closed conformation in insulin, IGF-I and IGF-II, respectively. The transition thresholds from the open to the wide-open conformation in the core opening were found to be approximately 11, 12 and 13 Å for insulin, IGF-I and IGF-II, respectively. These thresholds were measured by superimposing the MD simulation trajectories of the proteins in the complex structure of insulin bound to the IR to fit the receptor binding interface. The thresholds of the different conformations in the C domain opening were determined in a similar manner to the core opening.

Fig 5 shows substantial differences in the PDFs of the core opening in the insulin-family proteins. Using the PDF of insulin as a reference, IGF-I has a narrower distribution around the closed conformation with a higher probability, whereas IGF-II has a broader spread in its distribution of probability of each conformation. It is also evident from Fig 5 that the wide-open conformation is a rare event in both insulin and IGF-I, but is more likely to occur in IGF-II. Regarding the C domain opening (Fig 6), the IGFs also exhibit significant differences in their PDFs. IGF-I is seen to have a sharp, intense distribution centered in the closed conformation, while IGF-II displays a more extended distribution.

Using the PDF data, the total probabilities of the different conformations were calculated for the core and C domain openings in the insulin-like proteins and are presented in Table 1. The core opening probabilities indicate that while the closed conformation dominates in all the proteins, there is significant variation in their probabilities. IGF-I has the highest probability of being in the closed conformation (0.74), followed by insulin (0.64) and then IGF-II (0.55). Thus, the closed conformation of IGF-I is by far the most prevalent with its open and wide-open conformations occurring only 26% of the time. In contrast, the open and wide-open conformations occur 45% of the time for IGF-II, suggesting a much more active behavior compared to insulin and IGF-I. In particular, the probability of the occurrence of the wide-open conformation is approximately 3 times higher for IGF-II than that for insulin and IGF-I, where it is a relatively rare event. Thus, it is much more probable for IGF-II to attain the conformation that fits the receptor binding interface. The C domain opening probabilities in Table 1 also show substantial differences between the two IGFs. In the case of IGF-I, the C domain opening probabilities are similar to those for the core opening because the two motions are highly correlated (Fig 4). In IGF-II, the open conformation is dominated by the C domain opening with probability 0.48. The combination of the C domain open and wide-open conformations in IGF-II gives a total probability of 0.64, which is more than twice that of IGF-I (0.27). As observed earlier, the core and C domain openings are uncorrelated in IGF-II, and this results in substantial differences between the probabilities of the two openings.

**Table 1:** Probabilities of the different conformations of the insulin-family proteins for the core and C domain openings.

Conformation	Core opening			C domain opening		
	Insulin	IGF-I	IGF-II	Insulin	IGF-I	IGF-II
Closed/Inactive	0.64	0.74	0.55	-	0.73	0.36
Open/Active	0.31	0.20	0.30	-	0.22	0.48
Wide-open/Active	0.05	0.06	0.15	-	0.05	0.16

The probabilities for the different conformations of IGFs and insulin reveal the hyperactive nature of IGF-II compared to IGF-I and insulin. This hyperactive behavior of IGF-II, together with the much more prevalent closed conformation of IGF-I compared to insulin, may explain the ability of IGF-II, and not IGF-I, to bind to the IR-A with high affinity and selectivity.

**Fig 7:**
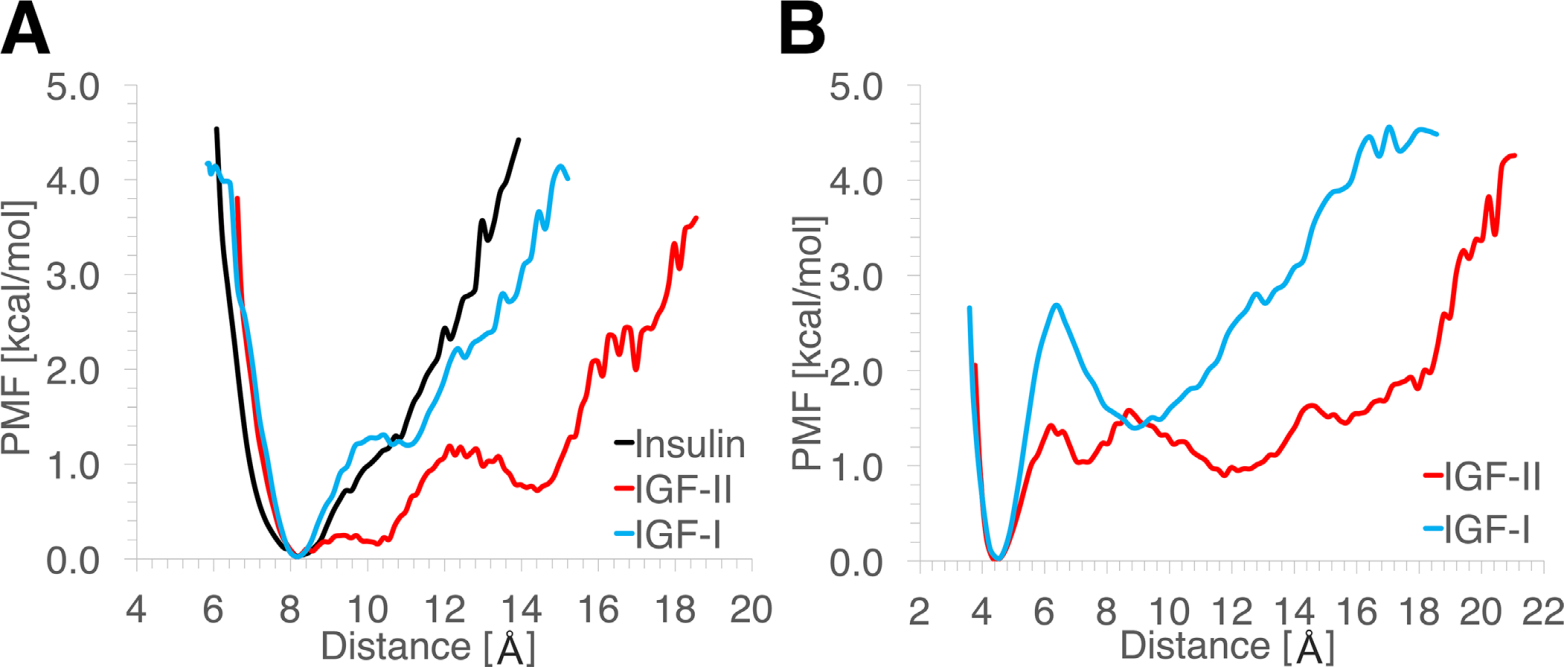
**PMFs calculated for (A) the core opening and (B) the C domain opening.** (A) Insulin, IGF-I and IGF-II are shown in black, blue and red, respectively. (B) IGF-I and IGF-II are shown in blue and red, similar to (A).

The energetics of the core and C domain openings in insulin and IGFs are shown in Fig 7. The PMFs were calculated based on the distance distributions. Because the PMFs are effective interactions, the equilibrium distances are relative. For ease of comparison, the curves in each plot are aligned at the same equilibrium distance and their minima are set to zero. Fig 7*A* compares the PMFs for the core opening in insulin and IGFs. IGF-II appears to be the most flexible–about 1 kcal/mol is needed to overcome the energetic barrier for the transition from the closed to the open conformation. Insulin and IGF-I show similar behavior, with the only difference being the initiation of the conformational change, which is seen to be energetically slightly more difficult in IGF-I. Fig 7*B* shows the energetics of the C domain opening in the IGFs. In IGF-I, the closed and open conformations are separated by a relatively large energy barrier, followed by a distinct energy well for the open conformation. In the core opening case (Fig 7*A*), there is only a hint of such a well. Thus, while the core and C domain openings are correlated, they exhibit qualitatively different behaviors with the latter showing a much sharper transition. There are three energetic barriers in the C domain opening of IGF-II (Fig 7*B*). From left to right, the first one corresponds to the transition of the C domain from the closed to the open conformation, while the core is in its closed conformation. The second barrier indicates the transition of the core to the open conformation while the C domain is in its open conformation. The third barrier corresponds to the transition of both the core and C domain openings to the wide-open conformation. Contrasting the energetics of opening, we observe that IGF-II needs only half the energy (≈ 1.4 kcal/mol) for the transition from the closed to the open conformation compared to IGF-I (≈ 2.8 kcal/mol). Furthermore, in IGF-II, the energy needed to overcome the other two energetic barriers is much smaller than the first one, indicating that the transition from the open to the wide-open conformation does not require much extra energy. In other words, the wide-open conformation of the C loop in IGF-II is as energetically favorable as the open conformation, thus justifying our description of the C loop as “hyperactive”.

### The critical role of the hinge and the hydrophobic interactions in the stability and activity of the insulin-family proteins

**Fig 8:**
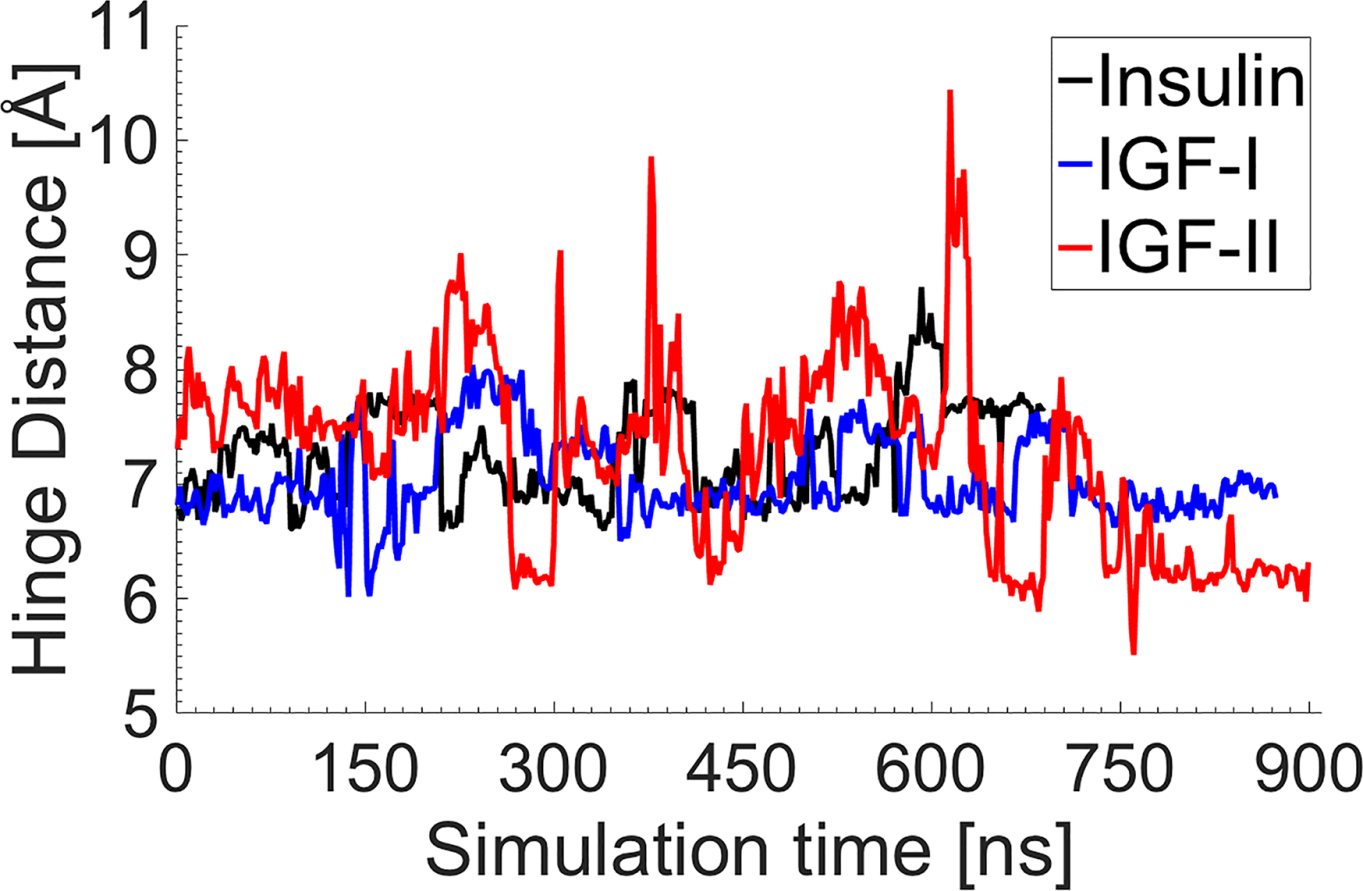
Hinge stability over the course of the MD simulations in insulin and IGFs. The hinge interaction involves the residues F24-L15, F23-L14 and F26-L17 in insulin, IGF-I and IGF-II, respectively. The hinge distance was calculated using the distance between the center of mass of the benzene ring of the hinge residue and the C_δ1_ atom of the leucine residue, which is the closest hydrophobic residue in the B domain α-helix.

The role of the F24 residue has been extensively studied in insulin, revealing its hinge-like role during the activation of insulin and its critical importance for high binding affinity to the IR [23–25, 29, 42] (cf. S3 Fig). To investigate the role of the hinge residue in IGFs and compare with insulin, the distances between the center of mass of the benzene rings of the F24/F23/F26 residues and the C_δ1_ atom of the L15/L14/L17 residues of the B domain α-helix were calculated over the course of the long MD simulations. These pairs represent the strongest interaction between the hinge residue and the residues of the hydrophobic core. Fig 8 shows this distance in each protein. While in insulin and IGF-I the hinge exhibits relatively stable behavior with small fluctuations over the course of the MD simulations, the hinge fluctuations are much more significant in IGF-II, suggesting that the hinge residue interacts weakly with the hydrophobic core and is not very stable. This lack of hinge stability in IGF-II is likely to affect the motion of the C loop, making it more flexible compared to IGF-I and insulin.

Table 2 lists the strongest interactions in the hydrophobic core in each protein. The hinge interaction, namely, F24-L15 in insulin, F23-L14 in IGF-I and F26-L17 in IGF-II, is stronger and more stable in insulin and IGF-I, compared to IGF-II, where the fluctuation is two and three times larger than that of IGF-I and insulin. This demonstrates the crucial role of the hinge in the stability of the insulin-family proteins. A weak hinge in IGF-II is likely to influence the hydrophobic core by weakening the interactions, leading to a less stable core and a more active C loop. In our previous study [23], the Y26F mutant insulin was found not to affect the activity of insulin, which corroborates experimental studies, where this mutation was found to lead to a compatible binding affinity with near WT affinity to the IR [43–45]. Therefore, having a different amino acid in the critical residue (Y26 in insulin, and F25 and F28 in IGF-I and IGF-II, respectively) does not seem to contribute significantly to the different behavior between insulin and IGFs. The only other variation in the hydrophobic core arises from the strength of the interactions between the critical residue and the other hydrophobic residues of the core. The distances in Table 2 indicate that the interactions in IGF-I and insulin are generally stronger than in IGF-II, with the smallest fluctuations occurring in IGF-I and the largest ones in IGF-II. The most noticeable difference among the three proteins occurs for the interactions between the critical residues (Y26/F25/F28) and the isoleucine residues in the first α-helix of the A domain (I_A_2/I43/I42), which is much stronger in IGF-I compared to insulin and IGF-II. Hence, the relatively weak hydrophobic interactions in the core in combination with the unstable behavior of the hinge in IGF-II is further indicative of a hyperactive protein, whereas the strong interactions in the core and the stable and strong hinge interaction in IGF-I is indicative of it being the most inactive protein among the insulin-family.

**Table 2:** Pair distances indicating the strongest interactions in the hydrophobic core of the insulin-family proteins.

Insulin			IGF-I			IGF-II		
Residues	Average Distance [Å]	Std [Å]	Residues	Average Distance [Å]	Std [Å]	Residues	Average Distance [Å]	Std [Å]
**F24-L15**^a^	4.6	0.4	**F23-L14**^a^	4.7	0.6	**F26-L17**^a^	5.0	1.2
**Y26-I**_**A**_**2**^b^	8.1	3.4	**F25-I43**^b^	5.4	1.2	**F28-I42**^b^	9.0	2.2
**Y26-V**_**A**_**3**^b^	7.1	2.5	**F25-V44**^b^	6.5	1.8	**F28-V43**^b^	7.4	2.7
**Y26-L11**^c^	7.0	2.8	**F25-L10**^c^	7.6	2.1	**F28-L13**^c^	9.7	3.5
**Y26-V12**^c^	5.6	2.4	**F25-V11**^c^	6.2	1.9	**F28-V14**^c^	7.1	3.3
**Y26-L15**^c^	6.5	2.1	**F25-L14**^c^	6.0	1.3	**F28-L17**^c^	7.6	2.6

^a^ The hinge interaction in the insulin-family proteins.

^b^ The interactions between the critical residue and the residues of the first α-helix of the A domain.

^c^ The interactions between the critical residue and the B domain α-helix.

The average distance and the standard deviation (Std) were calculated from the long MD simulations.

From these results we can conclude that the strength of the hydrophobic core, especially the interactions with the first α-helix of the A domain, and the stability of the hinge have a substantial influence on the stability and activity of the insulin-family proteins.

### Unraveling the activation mechanism

**Fig 9:**
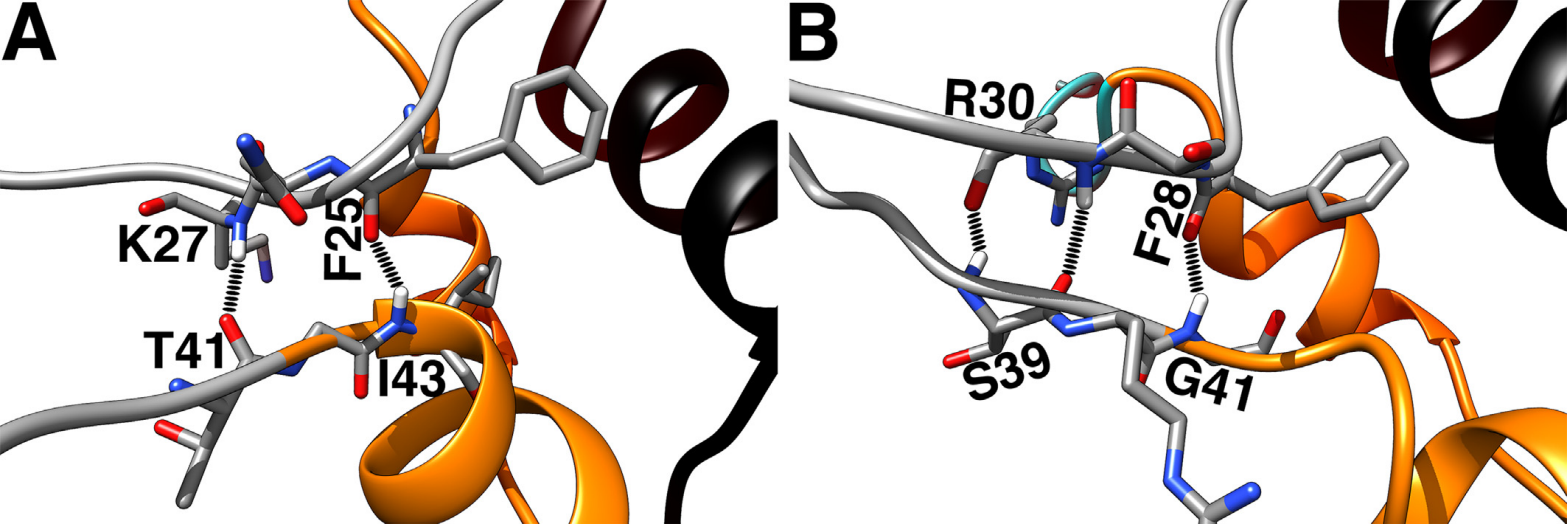
**Strongest hydrogen bonds between the upper flank of the C loop and its lower flank and/or A domain in (A) IGF-I and (B) IGF-II.** The hydrogen bonds are from left to right, (A) K27(N)-T41(O) and F25(O)-I43(N), and (B) R30(O)-S39(N), R30(N)-S39(O) and F28(O)-G41(N).

**Fig 10:**
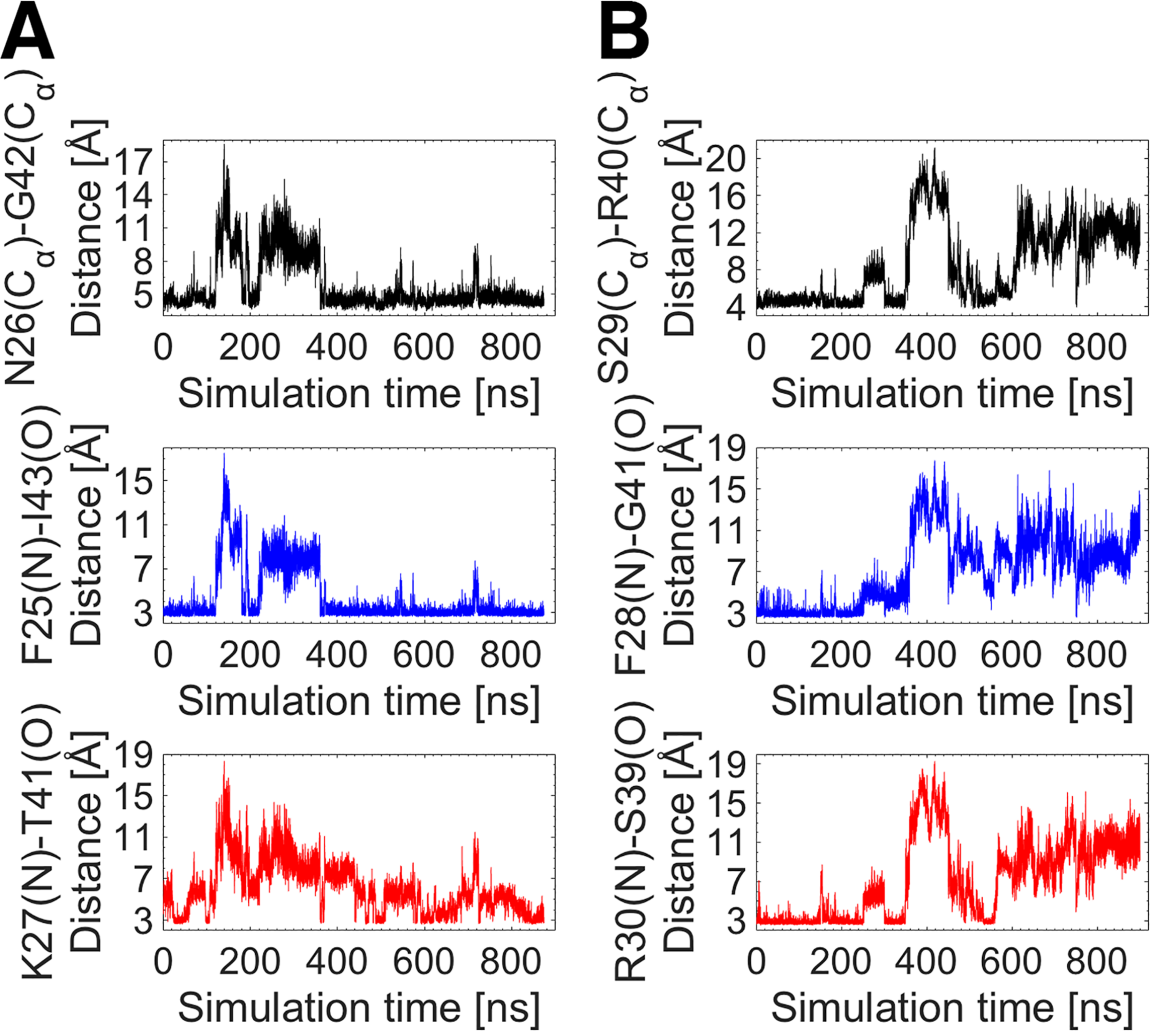
**Comparison of the C domain opening with the breaking of the strongest hydrogen bonds in (A) IGF-I and (B) IGF-II**.

IGFs have a network of hydrogen bonds between the upper flank of the C loop and its lower flank and/or the A domain. Many of these bonds are formed and broken over the course of the MD simulations. Fig 9 illustrates the strongest hydrogen bonds in IGFs. In both IGFs, a hydrogen bond exists between the critical residue and the first residues of the A domain, namely F25(O)-I43(N) in IGF-I and F28(O)-G41(N) in IGF-II. The other strong hydrogen bonds include K27(N)-T41(O) in IGF-I and a double hydrogen bond between the backbone atoms of the residues R30 and S39 in IGF-II, i.e. R30(O)-S39(N) and R30(N)-S39(O). Fig 10 shows the correlations between the breaking of these strong hydrogen bonds and the C domain opening in IGFs. In IGF-I, only the F25(O)-I43(N) hydrogen bond was found to be strong and stable over the course of the MD simulations, breaking only when the C domain opening occurs, as indicated by their correlated distance profiles in Fig 10*A*. In IGF-II, both the F28(O)-G41(N) and R30(N)-S39(O) hydrogen bonds exhibit stable behavior when the C loop is in its closed state, but break with the occurrence of the C domain opening (Fig 10*B*), suggesting again a correlation between the breaking of the hydrogen bonds and the C domain opening.

**Fig 11:**
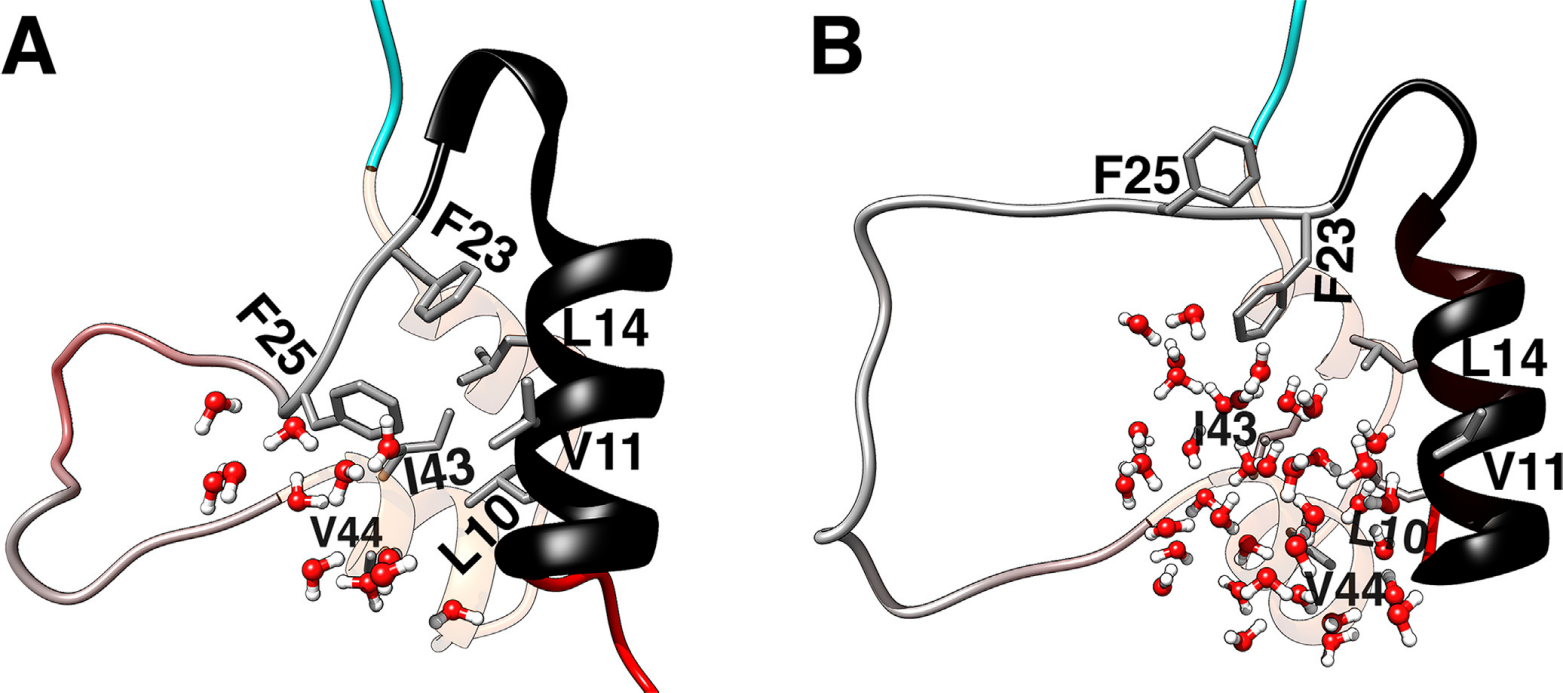
Illustration of the infiltration of water molecules into the hydrophobic core, breaking the hydrophobic interactions and triggering the activation of IGF-I. (A) Water molecules are located outside the lower extremity of the hydrophobic core when the protein is at the closed/inactive state; and (B) water molecules enter the hydrophobic core and break the interactions inside the core, thereby triggering the core opening.

**Fig 12:**
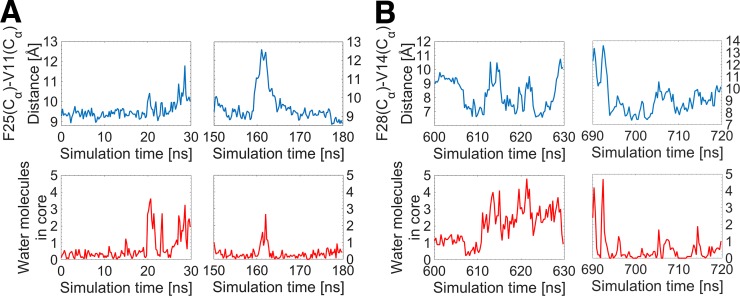
The entry of water molecules within the hydrophobic core as a trigger of the core opening in IGFs. The number of water molecules in the core (red) is correlated with the core opening (blue), for two randomly chosen intervals of the MD simulations of (A, B) IGF-I and (C, D) IGF-II.

In our previous study [23], we found that entry of water molecules into the hydrophobic core of insulin is responsible for triggering the BC-CT activation mechanism. Figs 11 and 12 shows that the activation mechanism of the IGFs is triggered in exactly the same way. In particular, when the C loop is in its closed/inactive state, water molecules are located outside the lower extremity of the hydrophobic core. The conformational change from the closed/inactive to the open/active state of the C loop occurs when water molecules randomly enter the core, weakening and breaking the interactions therein. Hence, the activation mechanism of IGFs is correlated with two events: i) the insertion of water molecules in the hydrophobic core, which breaks up the core interactions and initiates the core opening, and ii) the breaking of the strong hydrogen bonds in each IGF, triggering the C domain opening. Notably, the F25(O)-I43(N) and F28(O)-G41(N) hydrogen bonds in IGF-I and IGF-II, respectively, are seen to act as a lock for maintaining the proteins in their closed/inactive state by stabilizing the position of the critical residue in the hydrophobic core.

In conclusion, our results, obtained from long MD simulations, have demonstrated, for the first time, the similarities and differences in the activation mechanisms among the insulin-family proteins. We find that IGFs have an additional activation pathway via opening of the C loop, which has provided new insights into the affinities and specificities of these proteins to the IR-A. Although the IGFs show similar behavior to insulin in terms of the final conformation (wide-open) needed for receptor binding, we have found that the independent dynamics and energetics of the hydrophobic core and C domain in IGF-II are responsible for its observed substantially higher level of activity compared to IGF-I, where the core and C domain are coupled. Our results may thus contribute to the prediction and design of potential therapeutic antibodies to regulate the activity of IGFs, which are key cancer targets. For example, as the activity of the IGFs is closely correlated with the activity of the C loop and the hydrophobic interactions, a stronger hydrophobic core may lead to a less active IGF-II.

## Supporting Information

S1 Fig **Time series of the distances between the C_α_ atoms of Y26-V12, F25-V11 and F28-V14, which correspond to the criterion of the core opening in (A) insulin, (B) IGF-I and (C) IGF-II**.
(TIF)

S2 Fig **Time series of the distances between the C_α_ atoms of N26-G42 and S29-R40, which correspond to the criterion of the C domain opening in (A) IGF-I and (B) IGF-II**.
(TIF)

S3 Fig Representation of insulin in complex with the IR-A (L1 and αCT domains), as well as the hinge residue (F24) and its interactions with the B-chain α-helix (L15) and the L1 (L37 and F39) and αCT (F714) domains of the IR-A. Insulin B-chain is shown in black, Insulin BC-CT in blue, Insulin A-chain in orange, L1 domain in cyan, and αCT domain in pink.
(TIF)

S1 Table Sequence homology (in %) of the different domains of the insulin-family proteins. The percentages were calculated according to the smallest number of residues in each domain.
(XLSX)
