# Supplementary figures and images for: Elucidating the Activation Mechanism of the Insulin-Family Proteins with Molecular Dynamics Simulations
Source: PLoS One. 2016 Aug 22;11(8):e0161459. doi: 10.1371/journal.pone.0161459 (PMC4993506; doi:10.1371/journal.pone.0161459)

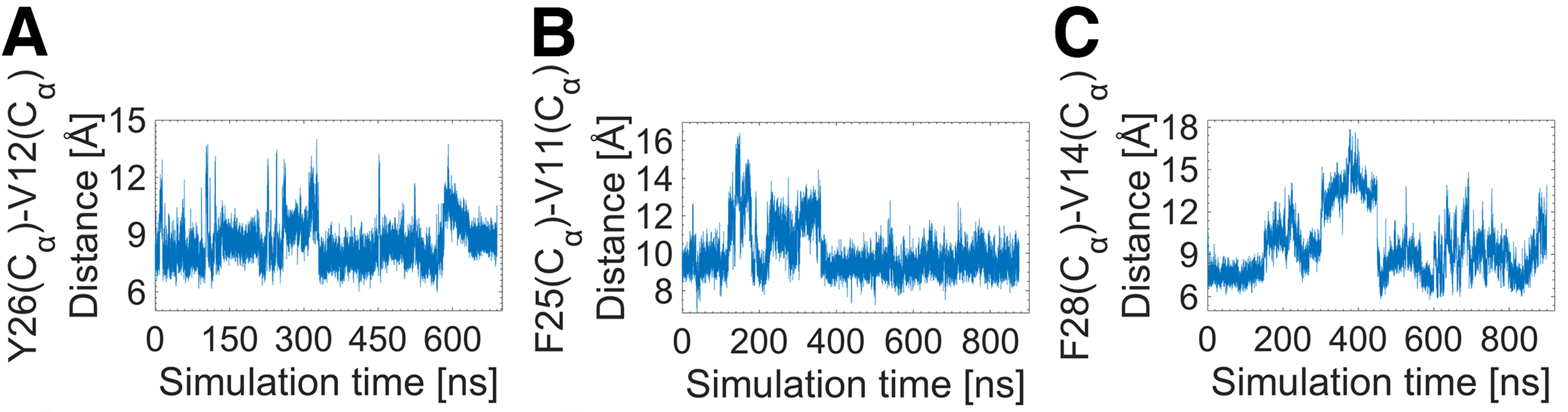

Supplement: S1 Fig — Time series of the distances between the Cα atoms of Y26-V12, F25-V11 and F28-V14, which correspond to the criterion of the core opening in (A) insulin, (B) IGF-I and (C) IGF-II. (TIF) [file pone.0161459.s001.tif]

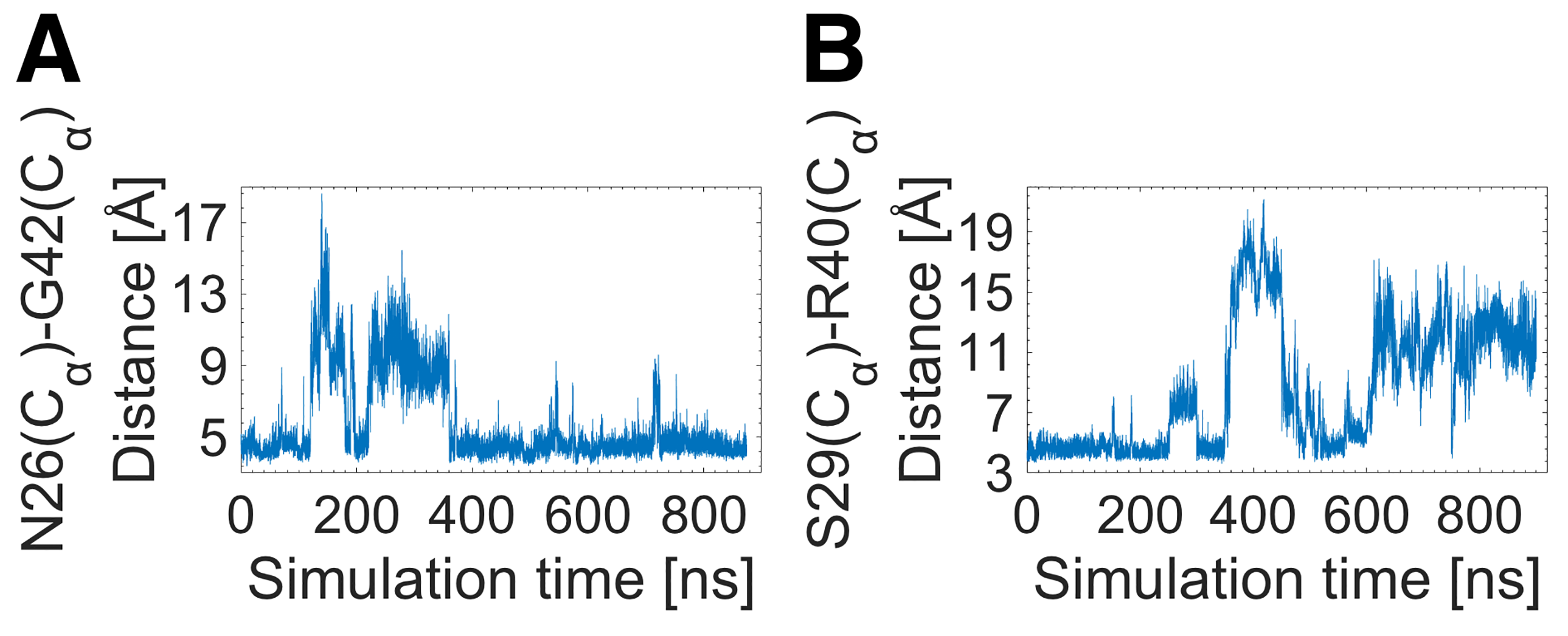

Supplement: S2 Fig — Time series of the distances between the Cα atoms of N26-G42 and S29-R40, which correspond to the criterion of the C domain opening in (A) IGF-I and (B) IGF-II. (TIF) [file pone.0161459.s002.tif]

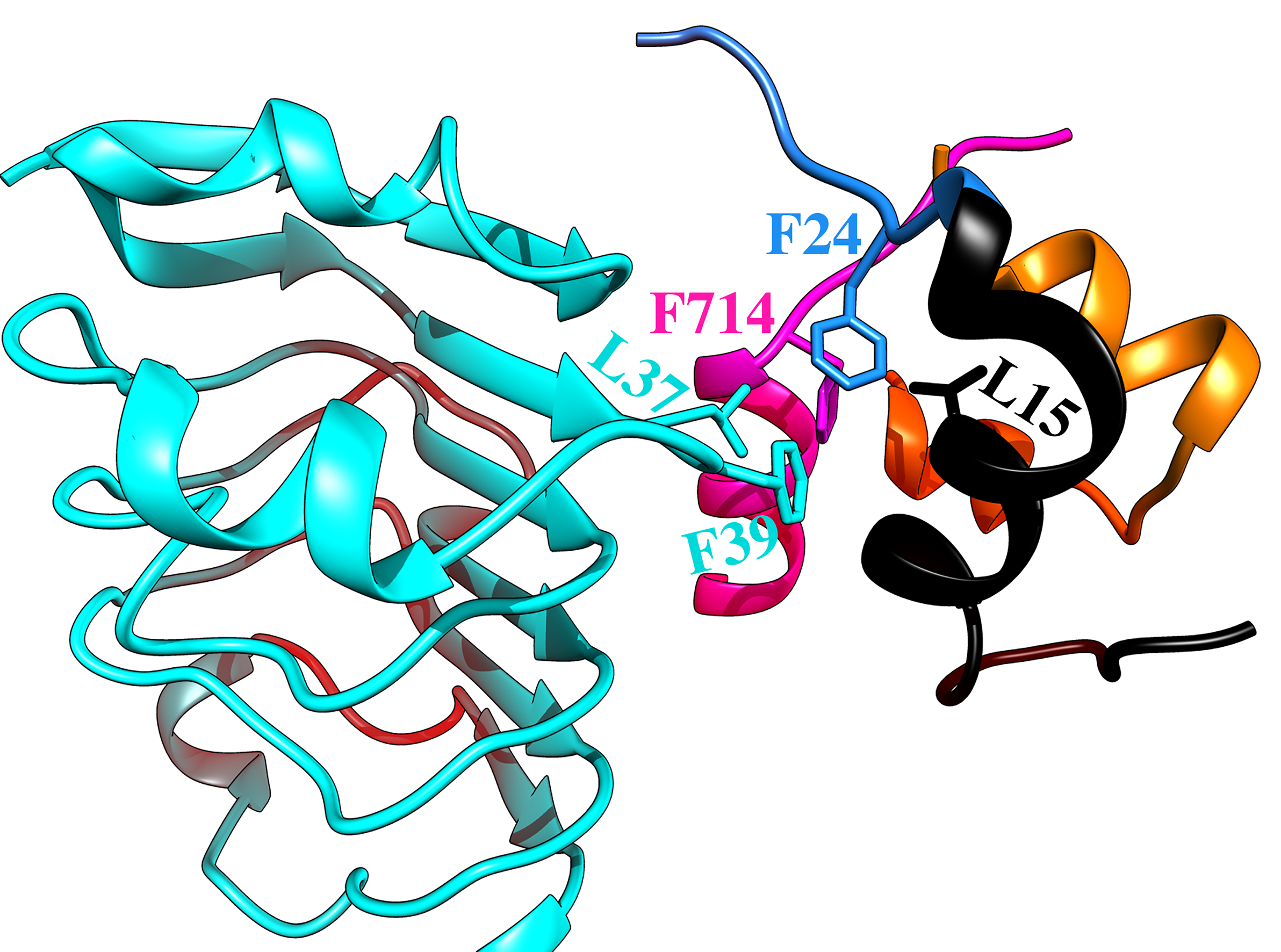

Supplement: S3 Fig — Insulin B-chain is shown in black, Insulin BC-CT in blue, Insulin A-chain in orange, L1 domain in cyan, and αCT domain in pink. (TIF) [file pone.0161459.s003.tif]
